# Just a consequence of hunger or a sign of chronic kidney disease in children? The dilemma of growth failure in countries with regional disparities

**DOI:** 10.1590/2175-8239-JBN-2024-E010en

**Published:** 2024-11-22

**Authors:** Marcelo de Sousa Tavares

**Affiliations:** 1 Santa Casa de Belo Horizonte, Belo Horizonte, MG, Brazil.

Growth failure is a hallmark of chronic kidney disease (CKD) in children^1^, and it has been a huge challenge in the treatment of childhood CKD. The etiology of growth failure in these patients is multifactorial. Reduced appetite, metabolic acidosis, dysregulation of the insulin-like growth factor 1 (IGF-1)/growth hormone axis, bone mineral disease, and anemia are among the main factors responsible for insufficient growth^2^. Different approaches, e.g. provision of an improved diet through gastrostomy/nasoenteral feeding, provision of alkali, recombinant human growth hormone, and metabolic control of bone mineral disease are the basis for optimizing growth in these children^1,2^. However, not all patients have access to all the resources that medicine provides for the treatment of this condition.

One of the main manifestations of CKD in childhood is reduced growth rate. In children regularly monitored by pediatricians in regions where the human development index and per capita income allow adequate child growth, growth retardation is easily recognized. On the other hand, in regions where per capita income is insufficient to ensure adequate nutrition and access to health care is compromised by factors that can range from distance to the place of care to the number of doctors per region or access to medications, prompt recognition of signs of CKD in the pediatric population, such as growth deceleration, will probably be more difficult to diagnose. Brazil is a country of continental dimensions and heterogeneous from a social and economic point of view. In the study conducted by Melo et al.^3^, children with CKD stages 3 to 5 who were not on dialysis and living in the state of Pernambuco, in the Northeast Region of Brazil, were analyzed regarding growth, its alterations, and associated factors. Some aspects of the study deserve attention, including the high proportion of urinary tract malformations (CAKUT) as the main etiology of CKD (88%), the median age range at the end of the study (7.9 years), and the progression in height at the end of follow-up (median of 36 months) without availability of recombinant growth hormone use. These findings reflect a profile observed in regions where the diagnosis of CKD in children is made late and where public resources for treatment are suboptimal, which may reflect an inefficient public health approach to the condition. A study by Konstantyner et al.^4^ revealed a panorama of children with CKD5 on dialysis in Brazil where the proportion of patients with CAKUT was lower in more economically developed areas of the country (southern regions). In 2020, the state of Pernambuco was ranked 24th among the 27 federative units in terms of per capita income, making it one of the lowest in Brazil^5^. The location where the study was conducted is a public institution, centralizing the treatment of pediatric patients with chronic kidney disease in the state. Consequently, we can assume that children with CKD referred to that institution were likely diagnosed and treated later than optimal. Similarly, the growth retardation associated with unrecognized chronic kidney disease in children may have been indirectly due to socioeconomic factors faced by the population of this state. Nascimento and Rodrigues^6^ reviewed the literature related to the nutritional status of children and adolescents in the northeast region of Brazil between 2009 and 2018. The authors found that there was a trend towards overweight and obesity throughout the period analyzed and that the highest rate of malnutrition was observed in Pernambuco^6,7^. The importance of the study by Melo et al.^3^ lies not only in the description of nutritional status, height progression and factors associated with growth retardation in that region, but also in the message to health teams to be aware of the potential diagnosis of CKD, especially when it is associated with urinary tract infection. Finally, the detection of pediatric cases of chronic kidney disease in resource-limited regions remains a major challenge.
