# Supplementary material for: Just a consequence of hunger or a sign of chronic kidney disease in children? The dilemma of growth failure in countries with regional disparities
Source: J Bras Nefrol. 2024 Nov 22;46(4):e2024E010. doi: 10.1590/2175-8239-JBN-2024-E010en (PMC11588343; doi:10.1590/2175-8239-JBN-2024-E010en)
Supplement: Translation

# Translation: Apenas uma consequência da fome ou um sinal de doença renal crônica em crianças? O dilema do retardo de crescimento em países com disparidades regionais

**DOI:** 10.1590/2175-8239-JBN-2024-E010pt

**Authors:** Marcelo de Sousa Tavares

**Affiliations:** 1 Santa Casa de Belo Horizonte, Belo Horizonte, MG, Brasil.

A retardo de crescimento é uma marca registrada da doença renal crônica (DRC) em crianças^1^. Tem sido um enorme desafio a ser superado no tratamento da DRC na infância. A etiologia da falha de crescimento nesses pacientes é multifatorial. A redução do apetite, acidose metabólica, desregulação do eixo do fator de crescimento insulina-símile 1 (IGF-1)/hormônio de crescimento, doença mineral óssea, anemia estão entre os principais fatores responsáveis pelo crescimento insuficiente^2^. Abordagens distintas, como melhora da oferta de dieta por meio de gastrostomia/alimentação nasoenteral, oferta de álcalis, hormônio de crescimento humano recombinante e controle metabólico da doença mineral óssea, são a base da otimização do crescimento dessas crianças^1,2^. No entanto, nem todos os pacientes têm acesso a todos os recursos que a Medicina proporciona para tratamento dessa condição.

Uma das principais manifestações da DRC na infância é a redução da velocidade de crescimento. Em crianças acompanhadas regularmente por pediatras em regiões com níveis de índices de desenvolvimento humano e renda *per capita* que permitam o adequado crescimento da criança, o retardo de crescimento é facilmente reconhecível. Por outro lado, renda *per capita* insuficiente para a provisão de alimentos e acesso à saúde comprometido por fatores que podem variar desde distância do local de atendimento até número de médicos por região ou acesso a medicamentos dificultam o pronto reconhecimento de sinais de DRC na população pediátrica, como desaceleração do crescimento. O Brasil é um país de dimensões continentais e heterogêneo do ponto de vista social e econômico. No estudo conduzido por Melo et al.^3^, foram analisadas crianças com DRC estágios 3 a 5 não dialíticas residentes no estado de Pernambuco, na região Nordeste do Brasil, no tocante ao crescimento, suas alterações e fatores associados. Alguns aspectos chamam a atenção no estudo. A proporção de malformações do trato urinário (CAKUT) como principal etiologia da DRC é elevada (88%), a faixa etária mediana no fim do estudo (7,9 anos) e a progressão da estatura no fim do acompanhamento (mediana de 36 meses) sem disponibilidade do uso de hormônio de crescimento recombinante refletem um perfil observado em regiões onde o diagnóstico da DRC em crianças é feito tardiamente e com recursos públicos subótimos para tratamento, podendo refletir uma abordagem de Saúde Pública ineficiente quanto a essa condição. Estudo de Konstantyner et al.^4^ revelou um panorama de crianças com DRC 5 em diálise no Brasil cuja proporção de pacientes com CAKUT foi menor em áreas economicamente mais desenvolvidas do país (região Sul). No ano de 2020, o estado de Pernambuco foi ranqueado em 24º entre 27 unidades federativas quanto a *renda per capita*, sendo, portanto, um dos estados com a menor renda do Brasil^5^. O local onde foi conduzido o estudo é uma instituição pública, centralizando o tratamento de pacientes pediátricos com doença renal crônica no estado. Como consequência, podemos supor que crianças com doença renal crônica naquele ano que chegaram àquela instituição naquele estado foram provavelmente diagnosticadas e encaminhadas para o tratamento tardiamente. De forma semelhante, o retardo de crescimento associado à doença renal crônica na infância não ter sido reconhecido precocemente pode ter sido motivado indiretamente por fatores socioeconômicos aos quais a população daquele estado é submetida. Nascimento e Rodrigues^6^ revisaram a literatura referente ao estado nutricional de crianças e adolescentes na região Nordeste do Brasil, publicada entre 2009 e 2018. Os autores constataram uma tendência à mudança em direção ao sobrepeso e obesidade ao longo do período analisado. A maior taxa de desnutrição foi observada em Pernambuco^6,7^.

A importância do estudo de Melo et al.^3^ está ligada não somente à descrição do estado nutricional, progressão estatural e fatores associados ao retardo de crescimento naquela região, mas também como mensagem às equipes de saúde para se atentarem ao potencial diagnóstico dessa condição clínica, principalmente quando associadas a casos de infecção do trato urinário. Por fim, a detecção de casos pediátricos de doença renal crônica permanece um grande desafio em regiões com recursos limitados.
